# Experimental comparative assay of tensile resistance of greater saphenous vein from ankle and groin

**DOI:** 10.1590/1677-5449.190117

**Published:** 2021-06-25

**Authors:** Carlos Eduardo Del Valle, Marcio Miyamotto, Jorge Rufino Ribas Timi

**Affiliations:** 1 Universidade Federal do Paraná – UFPR, Hospital de Clínicas, Unidade de Cardiologia e Pneumologia, Curitiba, PR, Brasil.; 2 Pontifícia Universidade Católica do Paraná – PUC-PR, Curitiba, PR, Brasil.; 3 Hospital Universitário Cajuru – HUC, Serviço de Cirurgia Vascular e Endovascular, Curitiba, PR, Brasil.; 4 Instituto VESSEL de Aperfeiçoamento Endovascular, Curitiba, PR, Brasil.; 5 Hospital Nossa Senhora das Graças – HNSG, Serviço de Cirurgia Vascular e Endovascular Elias Abrão, Curitiba, PR, Brasil.; 6 Núcleo Integrado de Cirurgia Endovascular do Paraná – NICEP, Curitiba, PR, Brasil.

**Keywords:** saphenous vein, varicose veins, vascular system injuries, vascular surgical procedures

## Abstract

**Background:**

The great saphenous vein is used as patch material in several types of arterial reconstruction, including trauma and carotid and femoral endarterectomy. There have been reports of saphenous patch blowout, particularly of patches constructed with veins harvested from the ankle. There is a need for objective measurement of the resistance of saphenous vein tissues.

**Objectives:**

To measure the tensile strength of the great saphenous vein harvested at the ankle and groin and analyze the correlation between diameter and tissue strength.

**Methods:**

Venous samples were harvested during elective saphenous stripping in patients with symptomatic varicose veins. Only segments without reflux were included. Ten limbs from eight patients were studied, providing 20 samples in total. Venous segments were opened along their longitudinal axis and fitted to electronic traction assay equipment to obtain values for material maximum tension in kilograms-force per square centimeter (kgf/cm^2^; the maximum force resisted by the segment, divided by its cross-sectional area).

**Results:**

The average maximum tension in the ankle saphenous vein group ranged from 74.02 to 190.10 kgf/cm^2^ and from 13.53 to 69.45 kgf/cm2 in the groin saphenous vein group (p < 0.0001). The Pearson coefficient for the correlation between vein diameter and maximum tension was -0.852 (moderate to strong inverse correlation).

**Conclusions:**

Ankle saphenous vein tissue from female patients operated for varicose veins has significantly higher resistance than saphenous vein tissue from the groin and there is an inverse relation between vein diameter and resistance of tissue from the same population.

## INTRODUCTION

The great saphenous vein can be used as patch material for closure of arteriotomy in many different anatomical territories, including for carotid endarterectomy,^1^^,^^2^ femoral endarterectomy,^3^ trauma care,^4^ and others. Use of patches for carotid endarterectomy has been studied extensively and is frequently associated with better results, both early and late,^5^^,^^6^ although some reports recommend primary closure.^7^^,^^8^ However, use of patches can be associated with certain complications, including infection^9^^,^^10^ and rupture.^11^^-^14 Rupture of a great saphenous patch after endarterectomy is a serious complication, with high rates of neurological sequelae and elevated mortality.^15^ The site (groin or ankle) from which great saphenous tissue is harvested for use in patching has been identified as a risk factor for rupture, with saphenous patches harvested from the ankle associated with cases of rupture.^3^^,^^12^ With the objective of assessing the resistance of great saphenous tissue harvested from different segments of the vein, this study compares the resistance of great saphenous vein tissue harvested during elective lower limb varicose vein surgery from segments free from reflux in the groin and ankle.

## METHOD

All patients were provided with free and informed consent forms, which they signed voluntarily. This study was submitted to and analyzed by the Human Research Ethics Committee at the Hospital de Clínicas da Universidade Federal do Paraná (UFPR), which granted preliminary approval under protocol number CEP/HC-UFPR 904.134/2004-08.

The inclusion criteria included the following:

- great saphenous veins intact in the region of the malleolus and close to the saphenofemoral junction (SFJ) in at least one of the lower limbs;
- total saphenectomy surgery planned to treat varicose veins;
- free from reflux in the regions under investigation;
- agreement to participation in the study, after receiving all due explanations from the study authors, being made aware of the risks and benefits involved, and reading and signing the free and informed consent form.

The exclusion criteria included the following:

- lower limbs that had undergone prior interventions involving the great saphenous vein region;
- lower limbs from which removal of both segments was not planned;
- presence of reflux documented with Doppler ultrasonography in the segments under investigation;
- veins with visible disease, such as phlebitis or excessive caliber change that would make their use for grafting unfeasible (less than 2 millimeters);
- age less than 18 years.

### Collection and transport of specimens

Segments of great saphenous vein were harvested from eight patients, two of whom underwent bilateral saphenectomy, making a total of ten lower limbs. From each limb, one segment was harvested from close to the SFJ and one segment was harvested at the ankle. Each segment had a minimum length of 3 centimeters. Specimens were harvested before the phleboextractor was used and, once removed, the operation was conducted as usual. Each segment was catheterized with a syringe and delicately distended with saline and then its mean diameter was measured in millimeters. The specimens were placed in chilled isotonic saline solution and transferred to the Experimental Surgery and Research Laboratory annexed to the Hospital for the tissue resistance tests.

### Traction texts

**Figure 1:**
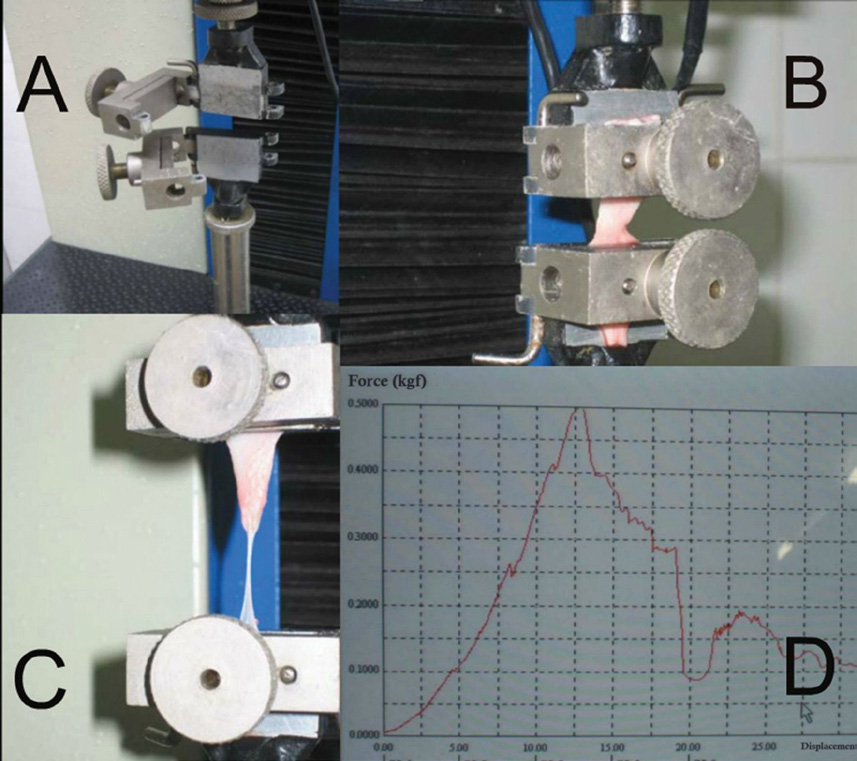
(A) Traction testing machine with grips; (B) venous segment cut open lengthwise, ready for use in the test; (C) segment after the traction test; (D) force against displacement graph provided by the test machine, illustrating the force applied as the vein is tractioned up to the point of maximum force resisted.

**Figure 2:**
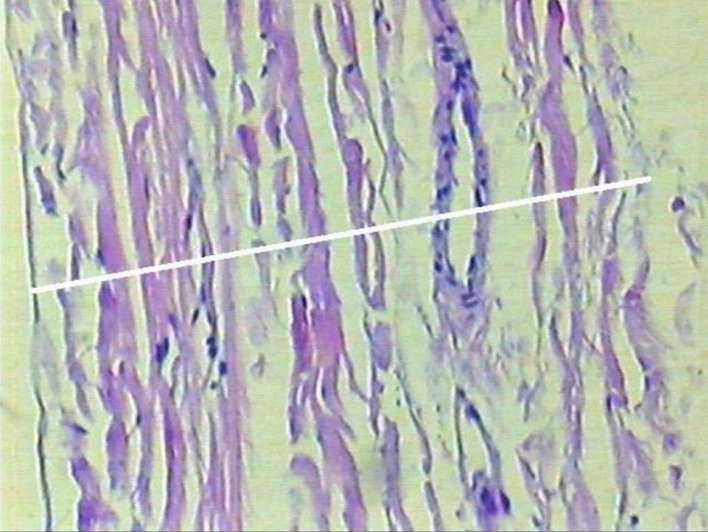
Measuring the thickness of the great saphenous vein under an optical microscope.

Tissue resistance was evaluated using an Instron 4467 computerized universal mechanical testing machine (Instron, London, UK),^2^^,^^16^ with pneumatic pressure grips and an electronic data acquisition system controlled by Instron IX software, version 7.26.00. Each venous segment was cut open lengthwise and its lateral extremities were fixed in the grips of the test machine for measurement (Figure 1). The machine then tractioned the tissue, plotting a graph of force against displacement and showing the maximum values resisted by the vein specimen in kilograms-force (kgf). The maximum tension resisted by the tissue is calculated automatically, in kilograms-force per square centimeter (kgf/cm^2^), by dividing the maximum force resisted by the cross-sectional area of the specimen. The cross-sectional area of the vein was calculated by multiplying its width after it had been cut open lengthwise by its thickness. The width of the cut open vein was calculated using the formula for circumference (C = 2πR). The mean thickness of the great saphenous vein was determined by harvesting 10 additional great saphenous vein segments, applying the same inclusion and exclusion criteria and employing the same preparation steps. After dilation and measurement of the vein, a cross-sectional segment was sent for study by microscope. The vein wall was measured using a ruler specially designed for optical microscopy, under 40 times magnification (Figure 2).

The mean great saphenous vein thickness was then determined for use in the maximum tension calculation. The maximum tension resisted by each vein segment was calculated using the formula tension = force/area. Force was measured by the test machine and area was calculated by multiplying the width of each segment by the mean great saphenous vein thickness. The maximum values resisted by the venous segments were recorded, both as absolute maximum force in kilograms-force and as maximum tension in kilograms-force per square centimeter of cross-sectional area (kgf/cm^2^).

### Variables analyzed

The variables analyzed were age, vein diameter at both sites, maximum tissue force, maximum tissue tension, comparison between proximal and distal maximum tissue force, comparison between proximal and distal maximum tissue tension, correlation between diameter and maximum tissue force for the entire sample and separately for each site and correlation between maximum tissue tension for the entire sample and separately for each site.

### Analysis of the difference between proximal and distal tissue resistance

Results were analyzed using Student’s *t* test for paired samples to compare paired samples for each donor. The test employs the principle of differences between the measurements for each pair, calculating the mean difference and testing whether the mean difference was different from zero within a given confidence interval.^17^^,^^18^ A 95% confidence interval was adopted for ruling out the null hypothesis (p < 0.05).

### Analysis of the correlation between diameter and tissue resistance

The degree of association between diameter and tissue resistance was evaluated by calculating Pearson’s correlation coefficient.^17^^,^^19^ This coefficient varies from (+1) to (-1), where the value zero equates to no association. Positive values indicate positive correlations, by which the magnitude of one variable tends to increase when the other variable increases. Negative values indicate negative correlations, or a tendency for one measurement to be smaller when the other increases. The correlation is interpreted as very weak if the coefficient has a value from zero to 0.2; weak for coefficients from 0.2 to 0.4; moderate for coefficients from 0.4 to 0.7; strong for coefficients for 0.7 to 0.9; and very strong for coefficients with values larger than 0.9.

## RESULTS

Table 1 lists the results for age and great saphenous vein diameter. All of the patients were female, with a mean age of 45.5 years and standard deviation of ± 10.57. The mean diameter of veins harvested from the ankle was 3.25 mm and mean diameter at the groin was 7.32 mm.

**Table 1:** Age of patients and diameters of the great saphenous vein at both sites.

	**Age**	**Diameter at ankle (mm)**	**Diameter at groin (mm)**
Mean	45.50	3.25	7.32
Standard deviation	10.57	0.76	1.64

The maximum forces resisted by the distal and proximal great saphenous vein specimens are shown in Table 2. The mean maximum force for distal veins was 3.34 kgf, with a standard deviation of 0.52 kgf. For the vein specimens from the region of the SFJ, mean maximum force was 2.20 kgf, with a standard deviation of 0.95 kgf. The result of Student’s *t* test for the comparison between the two groups was p = 0.0044.

**Table 2:** Maximum force and maximum tension resisted by great saphenous vein specimens from the ankle and groin, paired by lower limb.

**Patient**	**Maximum force - ankle (kgf)**	**Maximum force – groin (kgf)**	**Maximum tension - ankle (kgf/cm^2^)**	**Maximum tension – groin (kgf/cm^2^)**
1	2.91	1.12	128.65	21.60
2 (Right)	3.49	3.37	148.12	35.75
2 (Left)	2.78	2.37	101.71	29.58
3	2.89	2.29	82.87	44.17
4	3.52	1.25	81.19	13.53
5	4.30	3.54	190.10	62.60
6 (Right)	3.00	1.21	74.02	16.25
6 (Left)	3.00	1.41	106.10	19.94
7	4.27	1.85	156.22	28.04
8	3.24	3.60	90.46	69.45
	p = 0.004428		p = 0.00006222	
	t = 3.7684		t = 7.0146	
	Mean difference: 1.14		Mean difference: 81.8527	

The comparison between the tissue resistance for the distal and proximal great saphenous specimens in terms of maximum tension is also shown in Table 2. Mean maximum tension for veins from the ankle was 115.94 kgf/cm^2^ with a standard deviation of ± 36.51 kgf/cm^2^. Mean maximum tension for veins from the SFJ region was 34.09 kgf/cm^2^, with a standard deviation of ± 18.22 kgf/cm^2^. The result of Student’s *t* test for the comparison between the two groups revealed a significant difference between them (p = 0.00006222).

### Correlation between tissue resistance and diameter

**Figure 3:**
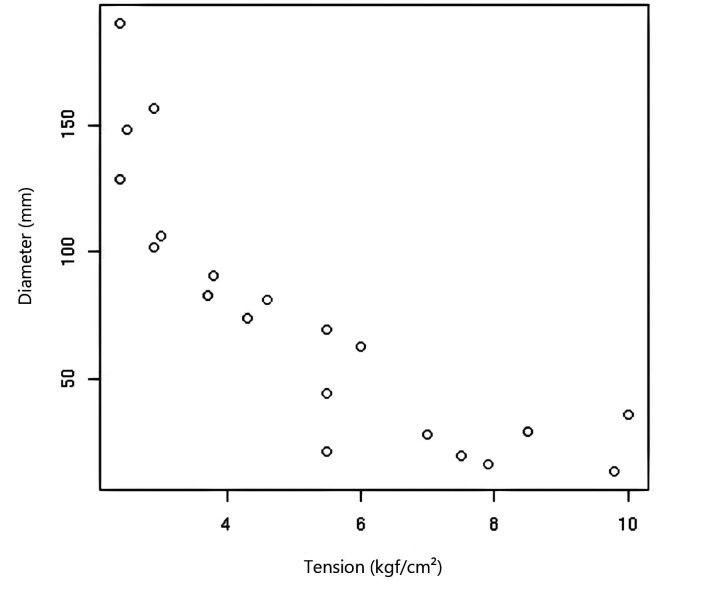
Correlation between maximum tension resisted by each segment of great saphenous vein and their respective diameters, for all 20 venous segments tested. Pearson’s correlation coefficient = -0.852247 (compatible with a strong inverse correlation between diameter and tissue resistance).

The correlation between the maximum tension resisted by the great saphenous vein specimens from both sites and their respective diameters is illustrated in Figure 3. The Pearson’s coefficient for this correlation was compatible with a strong inverse correlation between diameter and tissue resistance (p = -0.852247).

### Great saphenous vein thickness

The mean great saphenous vein thickness used to calculate maximum tension was 0.6 mm, with a standard deviation of ± 0.2 mm.

## DISCUSSION

Use of autogenous vein tissue to construct patches offers the advantages of better resistance to infection, reduced bleeding from suture orifices, an endothelialized and less thrombogenic surface, and lower cost, when compared to synthetic materials.^3^^,^^20^^,^^21^ The most common uses of autogenous vein patches include carotid endarterectomy, femoral endarterectomy, and trauma care.^3^^,^^4^^,^^21^

However, there are concerns with regard to the resistance of great saphenous tissue, because of reports of rupture of autogenous vein patches, both when used for carotid endarterectomy^11^^-^13 and when used for femoral endarterectomy,^3^^,^^22^ with invariably high mortality rates in both types of procedure. Rupture of autogenous vein patches has almost exclusively been described in relation to cases in which the vein was harvested from the ankle or leg, with no reports of rupture in patients who were treated with great saphenous vein patches harvested from the groin, with the exception of a single case in a questionnaire-based study by Tawes and Treiman^15^ Notwithstanding, the same study^15^ also reported ruptures of more distal vein tissues.

These events prompted several services to standardize use of great saphenous vein from the thigh or groin as the material of choice for carotid endarterectomy patching. Great saphenous vein diameter has been cited as a factor that should be taken into consideration when choosing the best segment to use as patch material, as in a case series reported by Archie in which only veins with diameter greater than 3.5 mm were used, harvesting the great saphenous vein from below the knee in 94% of male patients, none of whom suffered patch rupture.^12^ This approach was based on a study by Archie and Green who conducted experimental tests on specimens from great saphenous vein harvested during arterial reconstruction procedures, in which mean rupture pressure was no different for saphenous veins from the level of the ankle, knee, or SFJ.^23^ However, this methodological approach introduces selection bias, since veins from different levels are measured from different patients, including inter-individual variation as a confounding factor.

The design of the present study was planned to directly compare the tissue resistance of different segments of the great saphenous vein from each patient. This approach was intended to minimize the effect of inter-individual variation, using each patient as their own control. As a result, any differences found between mean tissue resistance for distal and proximal great saphenous material could not be attributed to differences in composition from one patient to another. The study only analyzed veins from patients operated to treat lower limb varicose veins. One of the inclusion criteria chosen was absence of reflux in the ankle and SFJ region, to avoid studying obviously pathological specimens. Thus, despite the bias introduced by analyzing patients with varicose veins, this was minimized by the fact that the specimens were identified as not diseased during preoperative mapping with Doppler ultrasonography. This approach meant that the rate of inclusion was low, since it was restricted to patients with long segmental reflux of the great saphenous vein who had indications for total saphenectomy, but did not have reflux in the regions that would be analyzed. The specimens collected were all analyzed within 2 hours of removal, to minimize the effect of time elapsed on the tissues.

The present study analyzed the tissue resistance of great saphenous vein specimens when tractioned in the longitudinal direction, because of the characteristics of the electronic test machine, which was unable to correctly secure the vein specimens in the transverse direction, because the minimum gap between its jaws was too large (Figure 1). Consequently, specimens with a length of 5 centimeters were harvested, enabling the experiment to be conducted. Donovan et al.^24^ compared tissue resistance in both directions (transverse and longitudinal), finding that longitudinal resistance was considerably greater, which is compatible with the descriptions of ruptures, which generally mention rupture along the transverse axis of the vein.^3^^,^^11^^,^^13^ This suggests that the risk of rupture would be greater when transverse traction is applied,^2^ unlike the experiment conducted in the present study. However, the present study involved paired analysis of specimens, to enable intra-patient evaluation of whether the vein material had greater resistance in one region than in another, to enable extrapolation of the comparison to other methodologies for measurement of tissue resistance.^2^ This derives from the fact that all methods for evaluation of the resistance of veins are simulations, since none of them will ever be capable of faithfully reproducing implantation of vein tissue as patches during surgery. Notwithstanding, measurement systems that in the future may be adapted to provide both longitudinal and transverse tension data could contribute to a more detailed assessment of the resistance of venous patches and grafts. Studies could also be developed that focus specifically on the plastic phase of the force against displacement graph, which is more representative of the structural stability of materials in general. The testing equipment used in the present study does not provide detailed information on the plastic phase, such as coefficient of rigidity or elastic limit.

Analysis of the maximum values in kilograms-force (kgf) resisted by the vein specimens revealed a statistically significant difference (p = 0.0044) between the ankle vein group and the groin vein group, with the distal veins resisting greater forces. However, this measurement does not take account of the diameter of the vessel or the thickness of its wall. A more precise idea of the resistance of the vein tissue is given by analyzing the magnitude of the maximum tension resisted, i.e. the total force resisted divided by the cross-sectional area. The cross-sectional area is the equivalent of the rectangle formed by the width of the vein after it has been cut open lengthwise and the thickness of its wall. The width of the vein after it has been cut open lengthwise is obtained from the diameter of the vein using the formula for circumference (C = 2πR). Dividing the force measured by the test machine by the cross-sectional area gives a value for the tension in kilograms-force per square centimeter (kgf/cm^2^). The thickness used for these calculations was the mean thickness obtained by microscopic measurements of a sample of ten different vein specimens, which was a mean of 0.06 mm. This option offered the advantage of eliminating the need to measure the thickness of each of the segments used in the traction tests under the microscope, but has the disadvantage that the tissue tension values could be distorted if there were large differences in the vein wall thickness of different specimens.

The tissue resistance measured in terms of maximum tension was significantly higher (p = 0.0000622) in the group of saphenous vein specimens harvested from the ankle compared with the specimens from close to the SFJ. The confidence interval was very high, which supports the conclusion that, even taking into account any imprecision caused by use of a fixed value for vein wall thickness, the distal saphenous specimens had higher resistance in this group of patients. With the reservation that the population analyzed in this study had clinically significant varicose veins, the findings suggest that using great saphenous vein tissue from the ankle region may not necessarily be contraindicated in all cases of arterial reconstruction. These data reveal a similar trend to one observed in a previous experimental study,^23^ i.e., saphenous veins harvested from the groin do not necessarily offer superior resistance to others. Additionally, the present study detected a strong negative correlation between diameter and tissue resistance (r = -0.85), suggesting that larger caliber veins may be less resistant. In the literature cited, the veins with lowest resistance to simulated intraluminal pressure were those with caliber less than 4 mm, which prompted the recommendation that small caliber veins should not be used as patch material. The discrepancy between the present study and the literature may be a result of the fact that the population analyzed here had varicose disease, which involves degeneration and weakening of the vascular wall and these disorders may affect different anatomic segments at different intensities. In the study by Van Damme et al.,^11^ one of the patients who suffered a central patch rupture had significant varicose veins in the contralateral limb, prompting the authors to recommend careful assessment of the macroscopic appearance of the vein, of the presence of significant lower limb varicose veins, and of the presence of signs of prior phlebitis in the vein to be used as patch material. These statements are compatible with our findings that the proximal great saphenous vein may not be the material that offers greatest resistance in patients with varicose veins.

The sample of specimens tested in this study were all harvested from female patients. The proportion of carotid endarterectomy patients who are female tends to oscillate around 40%.^25^^,^^26^ Previous studies of the tissue resistance of the great saphenous vein indicate that venous segments harvested from women have lower resistance. In practice, this corresponds to a higher prevalence of women in series reporting patch ruptures.^3^^,^^12^^,^^13^ The data presented here may therefore be of importance for the choice of site from where great saphenous vein material should be harvested for patching in women: in the case of patients who have varicose veins that are clearly detectable by clinical examination, using great saphenous vein material from close to the SFJ may involve risk and making this judgment based exclusively on diameter could be prone to failures. If the great saphenous vein at the ankle has a diameter greater than 3.5 mm, it would be the safer choice in these patients, if the criteria suggested in studies by Archie^12^^,^^23^ are applied in conjunction with the results of the analyses from this study.

## CONCLUSIONS

The results support the conclusion that the tissue resistance of great saphenous vein from the ankle is significantly higher than that of great saphenous vein harvested from close to the SFJ in female patients with lower limb varicose veins. There was a moderate inverse correlation between vein diameter and tissue resistance, in the same study population.

## References

[1] Edenfield L, Blazick E, Healey C (2019). Long-term impact of the Vascular Study Group of New England carotid patch quality initiative. J Vasc Surg.

[2] Miyamotto M, Del Valle CE, Moreira RCR, Timi JRR (2009). Comparative analysis of rupture resistance between glutaraldehyde-treated bovine pericardium and great saphenous vein. J Vasc Bras.

[3] Berner M, Lattmann T, Stalder P, Wigger P (2017). Vein patch closure using below the knee greater saphenous vein for femoral endarterectomy procedures is not always a safe choice. EJVES Short Reports..

[4] Moreira RCR, Del Valle CE, Thomaz JB, Belczak CEQ (2006). Trauma venoso.. Tratado de flebologia e linfologia..

[5] Bond R, Rerkasem K, Naylor AR, Aburahma AF, Rothwell PM (2004). Systematic review of randomized controlled trials of patch angioplasty versus primary closure and different types of patch materials during carotid endarterectomy. J Vasc Surg.

[6] Malas M, Glebova NO, Hughes SE (2015). Effect of patching on reducing restenosis in the carotid revascularization endarterectomy versus stenting trial. Stroke.

[7] Maertens V, Maertens H, Kint M, Coucke C, Blomme Y (2016). Complication rate after carotid endarterectomy comparing patch angioplasty and primary closure. Ann Vasc Surg.

[8] Chung BH, Heo SH, Park YJ, Kim YW, Woo SY, Kim DI (2020). Comparative analysis using propensity score matching analysis: primary closure versus patch angioplasty during carotid endarterectomy. Ann Vasc Surg.

[9] Fatima J, Federico VP, Scali ST (2019). Management of patch infections after carotid endarterectomy and utility of femoral vein interposition bypass graft. J Vasc Surg.

[10] Rizzo A, Hertzer NR, O’Hara PJ, Krajewski LP, Beven EG (2000). Dacron carotid patch infection: a report of eight cases. J Vasc Surg.

[11] Van Damme H, Grenade T, Creemers E, Limet R (1991). Blowout of carotid venous patch angioplasty. Ann Vasc Surg.

[12] Archie JP (1996). Carotid endarterectomy saphenous vein patch rupture revisited: selective use on the basis of vein diameter. J Vasc Surg.

[13] O’Hara PJ, Hertzer NR, Krajewski LP, Beven EG (1992). Saphenous vein patch rupture after carotid endarterectomy. J Vasc Surg.

[14] White SA, Thompson MM, Gaunt ME (1995). Vein patch rupture after carotid endarterectomy. Eur J Vasc Endovasc Surg.

[15] Tawes RL, Treiman RL (1991). Vein patch rupture after carotid endarterectomy: a survey of the Western Vascular Society members. Ann Vasc Surg.

[16] Ferreira M (2004). Radioterapia pré e pós-operatória na cicatrização de anastomoses colônicas em ratos avaliada mediante estudo tensiométrico, histológico e da morfometria do colágeno.

[17] Shimakura S, Shimakura S (2019). Comparação entre dois grupos.. Disciplina de Bioestatística CE008..

[18] Swinscow TDV, Campbell MJ (2019). The t tests. Statistics at square one..

[19] Swinscow TDV, Campbell MJ (2019). Correlation and regression. Statistics at square one..

[20] Louagie Y, Buche M, Eucher P, Goffinet JM, Laloux P, Jamart J (2011). Case-matched comparison of early and long-term outcomes of everted cervical vein and saphenous vein carotid patch angioplasty. Eur J Vasc Endovasc Surg.

[21] Muto A, Nishibe T, Dardik H, Dardik A (2009). Patches for carotid artery endarterectomy: current materials and prospects. J Vasc Surg.

[22] Flørenes T, Kroese A (1992). Rupture of the vein patch: a serious complication of profundaplasty. Eur J Surg.

[23] Archie JP, Green JJ (1990). Saphenous vein rupture pressure, rupture stress, and carotid endarterectomy vein patch reconstruction. Surgery.

[24] Donovan DL, Schmidt SP, Townshend SP, Njus GO, Sharp WV (1990). Material and structural characterization of human saphenous vein. J Vasc Surg.

[25] Chou EL, Sgroi MD, Chen SL, Kuo IJ, Kabutey NK, Fujitani RM (2016). Influence of gender and use of regional anesthesia on carotid endarterectomy outcomes. J Vasc Surg.

[26] Jim J, Dillavou ED, Upchurch GR (2014). Gender-specific 30-day outcomes after carotid endarterectomy and carotid artery stenting in the Society for Vascular Surgery Vascular Registry. J Vasc Surg.

