# Supplementary material for: Experimental comparative assay of tensile resistance of greater saphenous vein from ankle and groin
Source: J Vasc Bras. 2021 Jun 25;20:e20190117. doi: 10.1590/1677-5449.190117 (PMC8244984; doi:10.1590/1677-5449.190117)
Supplement: Translation

# Translation: Estudo experimental comparativo da resistência tensional da safena magna no tornozelo e na região inguinal

**Authors:** Carlos Eduardo Del Valle, Marcio Miyamotto, Jorge Rufino Ribas Timi

**Affiliations:** 1 Universidade Federal do Paraná – UFPR, Hospital de Clínicas, Unidade de Cardiologia e Pneumologia, Curitiba, PR, Brasil.; 2 Pontifícia Universidade Católica do Paraná – PUC-PR, Curitiba, PR, Brasil.; 3 Hospital Universitário Cajuru – HUC, Serviço de Cirurgia Vascular e Endovascular, Curitiba, PR, Brasil.; 4 Instituto VESSEL de Aperfeiçoamento Endovascular, Curitiba, PR, Brasil.; 5 Hospital Nossa Senhora das Graças – HNSG, Serviço de Cirurgia Vascular e Endovascular Elias Abrão, Curitiba, PR, Brasil.; 6 Núcleo Integrado de Cirurgia Endovascular do Paraná – NICEP, Curitiba, PR, Brasil.

**Keywords:** veia safena, varizes, lesões do sistema vascular, procedimentos cirúrgicos vasculares

## Abstract

**Contexto:**

A veia safena magna é usada como material de remendo em vários tipos de reconstrução arterial, incluindo no trauma e endarterectomias de carótida e femoral. Houve relatos de ruptura do remendo de safena, particularmente de veias colhidas na região do tornozelo. Há uma necessidade de medição objetiva da resistência tecidual da safena magna.

**Objetivos:**

Mensurar a força tensional suportada pela veia safena magna e analisar a correlação entre resistência e diâmetro da veia.

**Métodos:**

As veias foram coletadas durante operações de safenectomia por varizes dos membros inferiores. Foram analisados apenas segmentos sem refluxo. Foram analisados 10 membros de oito pacientes, com um total de 20 espécimes. Os espécimes foram submetidos a ensaio de tração em equipamento eletrônico, obtendo-se os valores de tensão máxima do material em quilogramas-força por centímetro quadrado (kgf/cm^2^; força máxima dividida pela área de secção transversa do segmento submetido à tração).

**Resultados:**

A tensão máxima suportada pela veia safena do tornozelo variou de 74,02 a 190,10 kgf/cm^2^, e a tensão máxima da veia safena da crossa variou de 13,53 a 69,45 kgf/cm^2^ (p < 0,0001). O coeficiente de correlação de Pearson entre o diâmetro da veia distendida e a tensão máxima suportada foram iguais a -0,852 (correlação inversa moderada a forte).

**Conclusões:**

A resistência tecidual da veia safena magna do tornozelo é maior do que a da crossa em mulheres submetidas a operação de varizes; há correlação negativa entre o diâmetro da veia e sua resistência tecidual nessa mesma população.

## INTRODUÇÃO

A veia safena magna pode ser usada como material de remendo (*patch*) no fechamento de arteriotomias em vários territórios, entre elas a endarterectomia de carótida^1^^,^^2^, endarterectomia de femoral^3^, no trauma^4^ e outras. O uso de remendos em endarterectomia de carótida já foi extensamente estudado e é frequentemente relacionado a melhores resultados tanto precoces como tardios^5^^,^^6^, apesar de alguns relatos favorecerem o fechamento primário^7^^,^^8^. No entanto, o uso de remendos pode apresentar complicações específicas, entre elas infecção^9^^,^^10^ e ruptura^11^^-^14. A ruptura de um remendo de safena magna após endarterectomia é uma complicação grave, com altos índices de sequelas neurológicas e elevadas taxas de óbito^15^. O local de coleta da safena magna para uso como remendo (região inguinal ou tornozelo) já foi apontado como fator de risco para ruptura, com safenas colhidas na região do tornozelo sendo associadas aos casos de ruptura^3^^,^^12^. Para avaliar a resistência tecidual da veia safena magna em seus diferentes segmentos, este estudo visa avaliar e comparar a resistência tecidual da veia safena magna em segmentos sem refluxo retirados da região inguinal e do tornozelo durante cirurgias eletivas de varizes dos membros inferiores.

## MÉTODO

Todos os pacientes tiveram acesso ao Termo de Consentimento Livre e Esclarecido (TCLE), assim como assinaram voluntariamente. O presente estudo foi apresentado, analisado e aprovado preliminarmente pelo Comitê de Ética em Pesquisa em Seres Humanos do Hospital de Clínicas da Universidade Federal do Paraná (UFPR), protocolo CEP/HC-UFPR 904.134/2004-08.

Entre os critérios de inclusão, estavam os seguintes:

- veias safenas magnas intactas tanto na região do maléolo como na região sua crossa em pelo menos um dos membros inferiores;
- plano cirúrgico de safenectomia total por varizes;
- ausência de refluxo nas regiões a serem estudadas;
- concordância em participar do estudo, após serem devidamente esclarecidos pelos autores, estarem cientes dos riscos e benefícios do mesmo e lerem e assinarem o TCLE.

Já entre os critérios de exclusão, constavam os seguintes:

- membro inferior submetido a qualquer intervenção no território da veia safena magna;
- membro inferior em que não estivesse planejada a coleta de ambos os segmentos planejados;
- presença de refluxo documentado pelo eco-Doppler nos segmentos a serem analisados;
- veia com doença visível, como flebite ou calibre excessivamente alterado a ponto de inviabilizar seu uso como enxerto (menor que 2 milímetros);
- idade menor que 18 anos.

### Coleta e transporte dos espécimes

Foram retirados segmentos da veia safena magna de oito pacientes, sendo que dois pacientes foram submetidos a safenectomia bilateral, totalizando dez membros inferiores. Em cada membro, foi colhido um segmento junto à crossa e outro no tornozelo, tendo cada segmento no mínimo 3 centímetros de extensão. O espécime foi coletado antes da passagem do fleboextrator pela região, e a operação foi, então, conduzida da maneira habitual. Cada segmento foi cateterizado com uma seringa, distendido delicadamente com soro fisiológico e teve seu diâmetro medido e registrado em milímetros. Os espécimes foram colocados em solução salina isotônica resfriada e levados para os testes de resistência tecidual, que foram realizados no Laboratório de Pesquisa e Cirurgia Experimental, anexo ao Hospital.

### Ensaio de tração

Para avaliação da resistência tecidual, foi utilizada máquina universal de ensaio mecânico computadorizada, da marca Instron, modelo 4467 (Instron, Londres, UK)^2^^,^^16^, com garras de pressão pneumática e sistema eletrônico de aquisição de dados com utilização do *software* Instron série IX, versão 7.26.00. Cada segmento venoso foi aberto no seu sentido longitudinal, e as extremidades laterais foram fixadas às presilhas da aparelhagem para medição (Figura 1). A máquina realiza a tração do tecido analisado, registrando em um gráfico a curva força *versus* deslocamento e proporcionando os valores máximos suportados pela veia em quilogramas-força (kgf). A tensão máxima suportada pelo tecido é dada automaticamente, em quilogramas-força por centímetro quadrado (kgf/cm^2^), através da divisão da força máxima suportada pela área de secção transversal da veia analisada. A área de secção transversal da veia foi obtida através da multiplicação da largura da veia aberta em seu sentido longitudinal pela sua espessura. A largura da veia aberta foi obtida através da fórmula do comprimento da circunferência (C = 2πR). Para a obtenção da espessura média da veia safena magna, foram colhidos mais dez espécimes de veia safena magna, seguindo-se os mesmos critérios de inclusão e exclusão e o mesmo sistema de preparo. Após a dilatação e medição da veia, um segmento transversal foi encaminhado para estudo de microscopia. A medida da parede venosa foi realizada utilizando-se régua especial para microscopia óptica, sob aumento de 40 vezes (Figura 2).

**Figura 1:** (A) Máquina de ensaio de tração com as presilhas; (B) segmento venoso aberto em sentido longitudinal pronto para o ensaio; (C) segmento após ensaio de tração; (D) gráfico força vs. deslocamento fornecido pelo sistema do aparelho, demonstrando a força aplicada à medida que a veia é tracionada até o ponto de máxima força suportada.

**Figura 2:** Medida da espessura da veia safena magna sob microscopia óptica.

Foi, então, calculada a espessura média da veia safena magna, para uso no cálculo da tensão máxima. A tensão máxima suportada por cada segmento venoso foi calculada através da fórmula tensão = força/área. A força foi aferida na máquina de ensaio, e a área foi o resultado da multiplicação da largura de cada segmento pela espessura média da veia safena magna. Foram registrados os valores máximos suportados pelos segmentos venosos, tanto em força máxima absoluta em quilogramas-força como em tensão máxima em quilogramas-força por centímetro quadrado de área transversa (kgf/cm^2^).

### Variáveis analisadas

As variáveis analisadas foram idade, diâmetro da veia em ambas as regiões, força tecidual máxima, tensão tecidual máxima, comparação entre a força tecidual máxima proximal e distal, comparação entre a tensão tecidual máxima proximal e distal, correlação entre diâmetro e força tecidual máxima em toda a casuística e separadamente para cada região e a correlação entre tensão máxima tecidual em toda a casuística e separadamente para cada região.

### Análise da diferença da resistência tecidual proximal e distal

Os resultados foram analisados segundo o teste *t* de Student pareado, visando à comparação entre amostras pareadas para cada indivíduo. O teste seguiu o princípio das diferenças entre as medições dentro de cada par, calculando a diferença média e testando se a diferença média é igual a zero dentro de um intervalo de confiança^17^^,^^18^. O intervalo de confiança para afastar a hipótese de nulidade foi estabelecido em 95% (p < 0,05).

### Análise da correlação entre diâmetro e resistência tecidual

O grau de associação entre diâmetro e resistência tecidual foi verificado através do cálculo do coeficiente de correlação de Pearson^17^^,^^19^. O coeficiente varia de (+1) a (-1), sendo que o valor zero equivale a não associação. Os valores positivos denotam correlação positiva, com uma variável tendendo a ter maior magnitude quando a outra aumenta. Os valores negativos denotam correlação negativa ou a tendência de uma medição ser menor à medida que a outra crescer. A correlação foi interpretada e atribuída como muito fraca no caso de coeficiente entre zero e 0,2; fraca para coeficiente entre 0,2 e 0,4; moderada para coeficiente entre 0,4 e 0,7; correlação forte para coeficiente entre 0,7 e 0,9; e muito forte para coeficiente acima de 0,9.

## RESULTADOS

Os resultados referentes a idade e diâmetro da veia safena magna podem ser vistos na Tabela 1. Todas as pacientes eram do sexo feminino, com média de idade de 45,5 anos com desvio padrão ± 10,57. O diâmetro médio das veias colhidas na região no tornozelo foi de 3,25 mm, e, na região inguinal, a média foi de 7,32 mm.

**Tabela 1:** Idade das pacientes e diâmetros da veia safena magna em ambas as regiões.

	**Idade**	**Diâmetro tornozelo (mm)**	**Diâmetro inguinal (mm)**
Média	45,50	3,25	7,32
Desvio padrão	10,57	0,76	1,64

A força máxima suportada pela veia safena magna distal e proximal está mostrada na Tabela 2. A força máxima para as veias distais atingiu a média de 3,34 kgf, com desvio padrão de 0,52 kgf. Para as veias provenientes da região da crossa, a média da força máxima foi de 2,20 kgf, com desvio padrão de 0,95 kgf. Para efeito comparativo entre os dois grupos, o teste *t* de Student pareado resultou em p = 0,0044.

**Tabela 2:** Força máxima e tensão máxima suportada pela veia safena magna da região do tornozelo e região inguinal, pareada por membro inferior.

**Paciente**	**Força máxima no tornozelo (kgf)**	**Força máxima inguinal (kgf)**	**Tensão máxima no tornozelo (kgf/cm^2^)**	**Tensão máxima inguinal (kgf/cm^2^)**
1	2,91	1,12	128,65	21,60
2 Direita	3,49	3,37	148,12	35,75
2 Esquerda	2,78	2,37	101,71	29,58
3	2,89	2,29	82,87	44,17
4	3,52	1,25	81,19	13,53
5	4,30	3,54	190,10	62,60
6 Direita	3,00	1,21	74,02	16,25
6 Esquerda	3,00	1,41	106,10	19,94
7	4,27	1,85	156,22	28,04
8	3,24	3,60	90,46	69,45
	p = 0,004428		p = 0,00006222	
	t = 3,7684		t = 7,0146	
	Média das diferenças: 1,14		Média das diferenças: 81,8527	

A comparação entre a resistência tecidual entre a safena magna distal e proximal através da medida da tensão máxima também está mostrada na Tabela 2. A média da tensão máxima para as veias provenientes do tornozelo foi de 115,94 kgf/cm^2^ com desvio padrão de ± 36,51 kgf/cm^2^, e a média da tensão máxima para as veias provenientes da região da crossa foi de 34,09 kgf/cm^2^, com desvio padrão de ± 18,22 kgf/cm^2^. Para efeito comparativo, o teste *t* de Student pareado mostrou diferença significativa entre os grupos (p = 0,00006222).

### Correlação entre resistência tecidual e diâmetro

A correlação entre a tensão máxima suportada pela safena magna em todos os segmentos e seus respectivos diâmetros está mostrada na Figura 3. Obteve-se coeficiente de correlação de Pearson compatível com forte correlação inversa entre diâmetro e resistência tecidual (p = -0,852247).

**Figura 3:** Correlação entre a tensão máxima suportada pela safena magna em cada segmento e seu respectivo diâmetro, para todos os 20 segmentos venosos submetidos ao experimento. Coeficiente de correlação de Pearson = -0,852247 (compatível com forte correlação inversa entre diâmetro e resistência tecidual).

### Espessura da veia safena magna

A espessura média da veia safena magna, para efeitos do cálculo da tensão máxima, foi de 0,6 mm, com desvio padrão de ± 0,2 mm.

## DISCUSSÃO

O uso de veia autógena para confecção de remendo (*patch*) tem como vantagens a melhor resistência a infecção, menor sangramento nos orifícios das suturas, superfície endotelizada e menos trombogênica e custo reduzido quando comparado aos materiais sintéticos^3^^,^^20^^,^^21^. Entre os usos mais comuns dos remendos de veia autógena, estão na endarterectomia de carótida, de femoral e no trauma^3^^,^^4^^,^^21^.

Existe, no entanto, uma preocupação quanto à resistência tecidual da safena magna, devido a relatos de ruptura de remendos de veia autógena tanto em endarterectomia de carótida^11^^-^13 como em endarterectomia de femoral^3^^,^^22^, sempre cursando com altas taxas de mortalidade em ambos os tipos de procedimento. A ruptura do remendo de veia autógena foi descrita quase que exclusivamente em casos em que a veia tenha sido retirada da região do tornozelo ou da perna, não havendo relato de rupturas em pacientes que receberam remendos de veia safena magna retirada da região inguinal, exceto um caso isolado no estudo baseado em questionários de Tawes e Treiman^15^. Porém, nesse mesmo estudo^15^, foram relatadas rupturas de veias mais distais.

Esses acontecimentos levaram vários serviços a padronizar o uso de safena magna da coxa ou região inguinal como escolha de material para remendo em endarterectomia de carótida. O diâmetro da veia safena magna foi citado como fator a ser considerado na decisão sobre o melhor segmento a se utilizar como remendo, como no caso relatado por Archie que, usando apenas veias com diâmetro maior que 3,5 mm, colheu a veia safena magna abaixo do joelho em 94% dos casos, e nenhum paciente apresentou ruptura^12^. Essa abordagem foi norteada pelo estudo de Archie e Green, em que a pressão média de ruptura não foi diferente na safena coletada na altura do tornozelo, do joelho ou da crossa^23^, fazendo o experimento em espécimes de veia safena magna coletados durante procedimentos de reconstrução arterial. Essa abordagem metodológica provoca um viés de seleção, ao medir veias de diferentes níveis em diferentes pacientes, incluindo a variação individual como fator de confusão.

O desenho do presente estudo planejou comparar diretamente em cada paciente a resistência tecidual da veia safena magna nos diferentes segmentos. Essa abordagem visou minimizar o efeito da variação individual, utilizando cada paciente como seu próprio controle. Sendo assim, não se poderia atribuir eventuais diferenças nas médias entre a resistência tecidual da safena magna distal e proximal à diferença de composição de um paciente para outro. Foram analisadas neste trabalho somente veias provenientes de pacientes operados de varizes dos membros inferiores. Optou-se por usar como critério de inclusão a ausência de refluxo na região do tornozelo e na região da crossa, para evitar o estudo de espécimes claramente patológicos. Assim, mesmo com o viés de serem analisados pacientes portadores de varizes, esse viés foi minimizado pelo fato de os espécimes não serem identificados como doentes no mapeamento pré-operatório com eco-Doppler. Com essa abordagem, a taxa de inclusão foi baixa, ficando restrita a pacientes com refluxo segmentar longo da veia safena magna e que tivessem indicação de safenectomia total, mas sem apresentar refluxo nas regiões que seriam analisadas no presente trabalho. Os espécimes coletados foram sempre analisados em até 2 horas sua retirada, para minimizar o efeito do tempo sobre os tecidos.

O presente estudo analisou a resistência tecidual da veia safena magna quando tracionada em seu sentido longitudinal, em virtude de o formato do aparelho eletrônico de medição não permitir a adequada preensão do vaso em seu sentido transversal devido à distância mínima muito grande entre as garras (Figura 1). Foi colhido, portanto, um segmento de 5 centímetros de comprimento do espécime, o que possibilitava a realização do ensaio. Donovan et al.^24^ compararam a resistência do tecido em ambos os sentidos (transversal e longitudinal), sendo a resistência longitudinal bem maior, fato compatível com as descrições de ruptura, que geralmente mencionam uma ruptura no sentido transversal da veia^3^^,^^11^^,^^13^. Isso sugere que o maior risco de ruptura é pela aplicação de tração transversal^2^, ao contrário do que foi avaliado no presente estudo. No entanto, o presente trabalho fez uma análise pareada entre os espécimes, visando justamente avaliar em cada paciente se o material que constitui a veia é mais resistente numa região do que na outra, para permitir a extrapolação da comparação para outras metodologias de aferição da resistência tecidual^2^. Isso vem do fato de que todos os métodos de avaliação de resistência da veia são simulações, posto que nenhum deles jamais terá a capacidade de reproduzir com total fidelidade o implante da veia como remendo em cirurgia. De qualquer forma, sistemas de mensuração que sejam no futuro adaptados para obter dados de tensão tanto longitudinal como transversal poderão contribuir para uma avaliação mais detalhada da resistência dos remendos e enxertos venosos. Também podem ser desenvolvidos estudos que ponham foco especificamente na fase plástica das curvas de força *versus* deslocamento, que são ainda mais representativas da estabilidade estrutural dos materiais em geral. No presente estudo, o equipamento utilizado não tinha capacidade de fornecer informações detalhadas da fase plástica, como coeficiente de rigidez e limite elástico.

A análise dos valores máximos em quilogramas-força (kgf) suportados pelas veias mostra uma diferença estatisticamente significante (p = 0,0044) entre o grupo de veias do tornozelo e o grupo de veias da região inguinal, com as veias distais suportando forças maiores. No entanto, essa medida não leva em conta o diâmetro do vaso nem a espessura de sua parede. Para se ter uma ideia mais precisa da resistência do tecido que compõe a veia, a grandeza a ser analisada é a tensão máxima, ou seja, a força suportada pela veia dividida pela área de sua seção transversa. Essa seção transversa equivale à área do retângulo cujos lados são a largura da veia aberta em seu sentido longitudinal e a espessura de sua parede. A largura da veia aberta em seu sentido longitudinal é obtida através da fórmula do comprimento da circunferência, a partir do diâmetro da veia (C = 2πR). Ao dividir-se a força medida no tensiômetro pela medida da sua seção transversa, será obtido o valor da tensão em quilogramas-força por centímetro quadrado (kgf/cm^2^). A espessura adotada nos cálculos foi a espessura média encontrada em medições microscópicas realizadas em outros dez espécimes, sendo essa espessura média de 0,06 mm. Essa opção trouxe consigo a vantagem de não ser necessária a utilização de medição microscópica da espessura de cada segmento submetido ao ensaio de tração, e a desvantagem reside na possível alteração dos valores de tensão tecidual em caso de grandes diferenças na espessura da parede vascular dos espécimes.

A resistência tecidual aferida pela tensão máxima foi significativamente maior (p = 0,0000622) no grupo de veias safenas provenientes da região do tornozelo em comparação com espécimes da região da crossa do vaso. O intervalo de confiança foi bastante alto, o que permite afirmar que, mesmo diante de imprecisões decorrentes da adoção de valor fixo para a espessura das veias, as safenas distais são mais resistentes nesse grupo de pacientes. Com a ressalva de que a população analisada neste trabalho era portadora de varizes clinicamente significativas, os presentes achados sugerem que o uso da veia safena magna proveniente da região do tornozelo pode não ser necessariamente contraindicado em todos os casos de reconstrução arterial. Os presentes dados mostram uma tendência similar àquela encontrada em um estudo experimental prévio^23^, ou seja, não há necessariamente uma superioridade na resistência das veias safenas provenientes da região inguinal em relação às outras. Além disso, no presente trabalho, foi encontrada uma forte correlação negativa entre diâmetro e resistência tecidual (r = -0,85), sugerindo que veias de maior calibre possam ser menos resistentes. Na literatura citada, as veias com a menor resistência à pressão intraluminal simulada foram aquelas com calibre menor que 4 mm, levando à recomendação de não se utilizar veias de pequeno calibre como material de remendo. Essa discrepância entre o presente trabalho e a literatura pode resultar do fato de a população aqui analisada ser portadora de doença varicosa, que cursa com degeneração e enfraquecimento da parede vascular, e essas alterações podem acometer os diferentes segmentos anatômicos com diferentes intensidades. No trabalho de Van Damme et al.^11^, um dos pacientes que sofreram ruptura central do remendo era portador de varizes significativas no membro contralateral, levando os autores a recomendar a avaliação cuidadosa do aspecto macroscópico da veia, da presença de varizes de membros inferiores significativas e da presença de sinais de flebite prévia na veia a ser usada como remendo. Essas afirmações vêm de encontro aos nossos achados, de que, em pacientes com varizes, a veia safena magna proximal pode não ser o material que apresenta maior resistência.

Na casuística deste trabalho, todos os espécimes foram retirados de pacientes do sexo feminino. Em endarterectomia de carótida, a parcela de pacientes do sexo feminino costuma flutuar ao redor de 40%^25^^,^^26^. Os estudos prévios de resistência tecidual da safena magna apontam para menor resistência nos segmentos venosos retirados de mulheres. Na prática, isso corresponde a uma alta prevalência de mulheres nas séries que relatam rupturas de remendo^3^^,^^12^^,^^13^. Assim, os presentes dados podem ser importantes na escolha do local de onde deve ser colhida a veia safena magna para remendo em mulheres: no caso da paciente ser claramente portadora de varizes detectáveis pelo exame clínico, pode ser arriscado utilizar a veia safena magna proveniente da região da crossa, e o julgamento baseado apenas no diâmetro pode apresentar falhas. No caso da veia safena magna do tornozelo com diâmetro maior que 3,5 mm, ela passa a ser a escolha mais segura nessas pacientes, se forem usados os critérios sugeridos por Archie em seus diversos estudos^12^^,^^23^, em conjunto com os resultados e as análises do presente trabalho.

## CONCLUSÃO

Os resultados demonstrados permitem concluir que a resistência tecidual da safena magna do tornozelo é significativamente maior do que a da safena magna da crossa em pacientes do sexo feminino com varizes dos membros inferiores. Há uma correlação inversa moderada entre o diâmetro da veia e sua resistência tecidual, nessa mesma população estudada.
